# Exploring the Molecular Mechanism of Action of Yinchen Wuling Powder for the Treatment of Hyperlipidemia, Using Network Pharmacology, Molecular Docking, and Molecular Dynamics Simulation

**DOI:** 10.1155/2021/9965906

**Published:** 2021-10-28

**Authors:** Jiahao Ye, Lin Li, Zhixi Hu

**Affiliations:** ^1^The Domestic First-Class Discipline Construction Project of Chinese Medicine, Hunan University of Chinese Medicine, Changsha, Hunan 410208, China; ^2^Institute of Traditional Chinese Medicine Diagnostics, Hunan University of Chinese Medicine, Changsha, Hunan 410208, China; ^3^Post-Graduate School, Hunan University of Chinese Medicine, Changsha, Hunan, China

## Abstract

**Background:**

Yinchen Wuling powder is often used to treat clinical hyperlipidemia, although its mechanism of action remains unclear. In this study, we aimed to investigate the active ingredients found in Yinchen Wuling powder and find its mechanism of action when treating hyperlipidemia, using a combination of network pharmacology, molecular docking, and molecular dynamics simulation approaches.

**Methods:**

The TCMSP database was used to obtain the principle active ingredients found in Yinchen Wuling powder and the NCBI and DisGeNet databases were used to obtain the main target genes involved in hyperlipidemia, and the intersectional targets were obtained by EXCEL. We also used Cytoscape 3.7.2 software to construct a “Traditional Chinese Medicine-Active Ingredient-Target” network and use STRING platform to conduct “protein-protein interactional” (PPI) analyses on the intersection targets. Bioconductor software and RX 64 4.0.0 software were then used to perform GO functional enrichment analysis and KEGG pathway enrichment analysis on the targets. Molecular docking of core protein-ligand interactions was modeled using AutoDock Vina software. A simulation of molecular dynamics was conducted for the optimal core protein-ligand obtained by molecular docking using Amber18 software.

**Results:**

A total of 63 active ingredients were found in Yinchen Wuling powder, corresponding to 175 targets, 508 hyperlipidemia targets, and 55 intersection targets in total. Cytoscape 3.7.2 showed that the key active ingredients were quercetin, isorhamnetin, taxifolin, demethoxycapillarisin, and artepillin A. The PPI network showed that the key proteins involved were AKT1, IL6, VEGFA, and PTGS2. GO enrichment analysis found that genes were enriched primarily in response to oxygen levels and nutrient levels of the vesicular lumen and were associated with membrane rafts. These were mainly enriched in AGE-RAGE (advanced glycation end products-receptor for advanced glycation end products) signaling pathway in diabetic complications, fluid shear stress, and atherosclerosis, as well as other pathways. The molecular docking results indicated key binding activity between PTGS2-quercetin, PTGS2-isorhamnetin, and PTGS2-taxifolin. Results from molecular dynamics simulations showed that PTGS2-quercetin, PTGS2-isorhamnetin, and PTGS2-taxifolin bound more stably, and their binding free energies were PTGS2-quercetin -29.5 kcal/mol, PTGS2-isorhamnetin -32 kcal/mol, and PTGS2-taxifolin -32.9 kcal/mol.

**Conclusion:**

This study is based on network pharmacology and reveals the potential molecular mechanisms involved in the treatment of hyperlipidemia by Yinchen Wuling powder.

## 1. Introduction

Hyperlipidemia represents a major global health problem and is caused by abnormal fat metabolism or its transport throughout the body, resulting in a variety of lipid structural disorders in plasma. Hyperlipidemia is generally caused by elevated levels of total cholesterol (TC), triglycerides (TG), and low-density lipoprotein cholesterol (LDL-C) and a reduction in high-density lipoprotein cholesterol (HDL-C) and represents one of the major risk factors for cardiovascular diseases [1]. Although antihyperlipidemic drugs have a rapid lipid-lowering effect, their side effects are dangerous as they can cause liver toxicity and rhabdomyolysis [2].

Traditional Chinese medicine has accumulated a wealth of experience in the regulation of blood lipids during its long practical applications. Yinchen Wuling powder (YCWL) is a classical prescription medicine used clinically to treat hyperlipidemia, and its use was first recorded in “Synopsis of Prescriptions of the Golden Chamber” and consists of Artemisia capillaris herba, Alismatis rhizoma, Atractylodes lancea, Polyporus umbellatus, Poria, and Cinnamomi ramulus [3, 4].

In recent years, there have been increasing clinical reports and experiments involving the treatment of hyperlipidemia with Yinchen Wuling powder; Zhang [5] reports the use of YCWL to treat hyperlipidemia and found that it could not only improve patient symptoms but also effectively regulate blood lipids, with few adverse side effects and therefore enjoyed high patient compliance. Ma and Li [6] also reported that YCWL in combination with low-dose simvastatin could not only effectively regulate blood lipids but also improve the clinical symptoms of patients undergoing maintenance hemodialysis. Li et al. [7] found using *in vivo* studies that YCWL can lower TCs, TGs, and LDL-Cs and increase the levels of HDL-C and improve hemodynamics and coagulation. Wu et al. [8] found that YCWL can also regulate the expression of LDL-R at the liver cell membrane when treating hyperlipidemia by regulating the levels of p38 MAPK and p42/44 MAPK and their phosphorylation status.

Modern pharmacological studies have shown that the scoparone and p-hydroxyacetophenone contained in the Artemisia capillaris herba play an important role in the process of regulating lipids, and scoparone contains peroxisome proliferators. Scoparone can antagonize peroxisome proliferator-activated receptors (PPARs), inhibit the formation of 3T3L1 preadipocytes, downregulate the expression of fat synthesis genes, and play a role in regulating fat metabolism [9]. p-hydroxyacetophenone can promote bile excretion and improve digestion, thereby controlling blood lipid levels, preventing cardiovascular complications, and regulating the physical function of patients with hyperlipidemia [10, 11]. The pachymic acid contained in Poria can induce the expression of glucose transporter 4 (GLUT4), stimulate the redistribution of GLUT4 from intracellular vesicles to the plasma membrane of adipocytes, and upregulate insulin receptor substrate 1 (IRS-1), Akt, and the phosphorylation level of AMP-activated kinase (AMPK). Pachymic acid can also induce the accumulation of triacylglycerol and inhibit lipolysis in differentiated adipocytes [12]. The cinnamaldehyde contained in Cinnamomi ramulus can downregulate blood glucose by upregulating the expression of GLUT4 gene level in mouse skeletal muscle [13]. Cinnamaldehyde enhances the antioxidant defense against reactive oxygen species produced under hyperglycemic conditions, thereby protecting pancreatic *β* cells from its loss and exhibiting antidiabetic properties. Liu et al. [14] found that that the polyporus polysaccharide contained in Polyporus umbellatus has a certain preventive and therapeutic effect on alcoholic fatty liver, and the polyporus polysaccharide can significantly reduce the cholesterol and triacylglycerol content of serum in rats with fatty livers and significantly increase the level of HDL-C and improve the degree of fatty degeneration of liver cells. An important part of the medicinal ingredients of Atractylodes lancea is volatile oil, which is mainly composed of atractylenolide, atractylone, and atractylol [15]. Studies have found that Atractylodes lancea volatile oil can effectively reduce the levels of TG, TC, fasting insulin (FINS), fasting blood glucose (FBG), and LDL-C in the serum of rats with metabolic syndrome and improve their insulin sensitivity and HDL-C content [16]. The alcohol and water extracts contained in Alismatis rhizoma are effective at lowering blood lipids, which can significantly reduce the serum TC, TG, and LDL-C levels of high-fat diet rats, increase the concentration of HDL-C, and promote the secretion of apo B in the liver. It can also reduce HMG-CoA reductase activity in a dose-dependent manner in vivo and in vitro [17–20].

Network pharmacology is a method for predicting the composition of the effective components and disease targets of a compound at the systems level and the establishment of a multilevel network such as drugs-components-targets-disease. This then enables locking the key targets related to drugs and diseases, using bioinformatics methodology. The target point corresponds to the new method of the pathway [21, 22]. Molecular docking uses flexible and semiflexible docking to evaluate the receptor-ligand interaction force, thereby predicting the receptor-ligand binding mode and affinity [23]. Molecular dynamics allows the simulation of various ligand and receptor motions through Newtonian mechanics to assess stability and flexibility.

**Figure 1:**
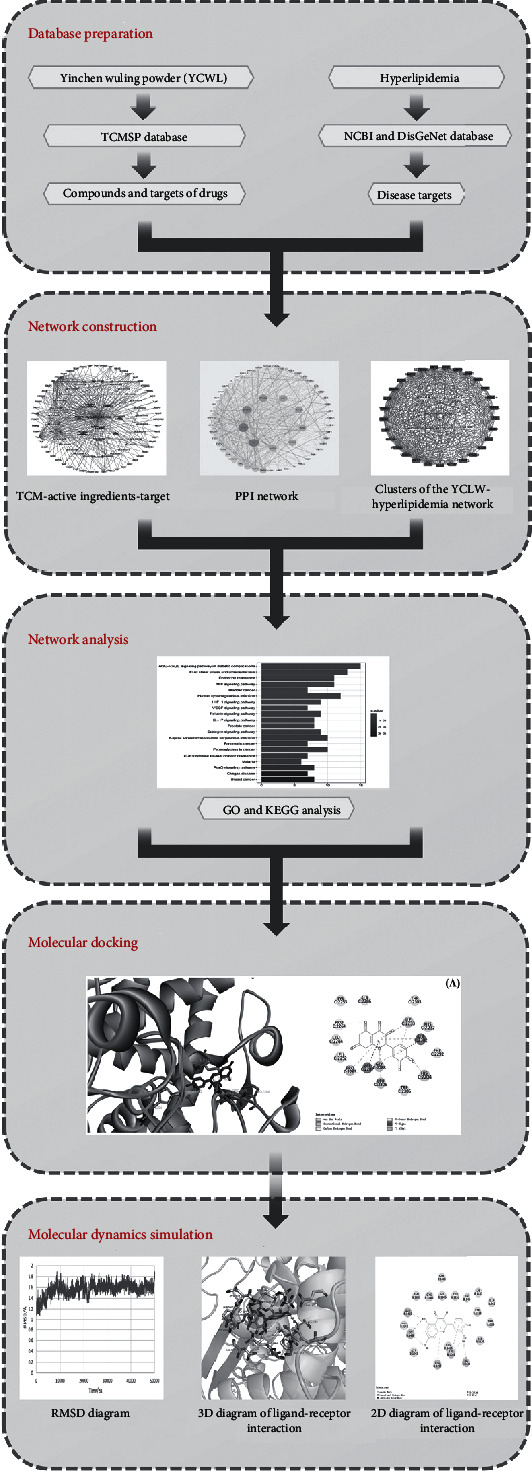
Workflow chart of YCWL for the potential treatment of hyperlipidemia based on network pharmacology.

This experimental study uses network pharmacology molecular docking and molecular dynamics simulation technology to explore the relevant biological pathways associated with YCWL in treating hyperlipidemia. It achieves this by constructing a drug and disease target network and then verifying it through molecular docking technology thus providing a theoretical basis for clinical application. A workflow chart is shown in Figure 1.

## 2. Method

### 2.1. Screening of Active Ingredients and Target Genes for YCWL

The active ingredients within YCWL were obtained from the TCMSP database (http://lsp.nwu.edu.cn/tcmsp.php), and the active ingredients were screened according to oral bioavailability (OB) ≥ 30% and drug‐likeness (DL) ≥ 0.18. The protein targets for the active ingredients were determined using the TCMSP platform [24].

### 2.2. Screening for Hyperlipidemia Targets and “Chinese Medicine-Disease” Intersection Targets

Using the NCBI (https://www.ncbi.nlm.nih.gov/) and DisGeNet (https://www.disgenet.org/) databases, we screened targets related to hyperlipidemia, and “hyperlipidemia” was entered as the search keyword, and the gene targets retrieved were a result of two merged disease databases, and all duplicate values were deleted. After the disease target was obtained, both the disease targets and the drug targets were combined to screen out common targets and construct a “Chinese medicine-disease” intersectional target database, which was visualized using a Venn diagram (https://bioinfogp.cnb.csic.es/tools/venny/index.html).

### 2.3. Constructing a “Traditional Chinese Medicine-Active Ingredient-Target” Network

Using Cytoscape 3.7.2 (http://cytoscape.org/), a network of “Traditional Chinese Medicine-Active Ingredients-Target” for YCWL was constructed to help determine its pharmacological mechanisms of action. The degree value reflects the importance of the node in the network; the higher the value, the more important the node. And the core active ingredients were screened out by the degree [25].

### 2.4. PPI Network Construction and Cluster Analysis

The intersection targets of Chinese medicine and disease were imported into STRING 11.0 database (https://string-db.org) to construct a PPI network model using biological species set to “Homo sapiens”; the confidence > 0.4 and unconnected nodes were hidden. The rest of the parameters are set to default values. From this, a protein-protein interaction network (PPI) was obtained [26] and the TSV file format was downloaded to construct a PPI network with Cytoscape 3.7.2 software; the network in STRING is the PPI. Some target proteins in cell biology activities are closely related and have the same or similar functions; these target proteins can be considered a cluster. Proteins in the same cluster are generally considered to play a synergistic role in disease progression. We use the MCODE plug-in in Cytoscape 3.7.2 to perform cluster analysis on protein targets in complex bioinformatics networks [27, 28] .

### 2.5. GO Enrichment Analysis

Using Bioconductor software and RX 64 4.0.0, the common targets of YCWL and hyperlipemia were analyzed for GO functional enrichment analysis. GO enrichment analysis selects three modules: biological process (BP), molecular function (MF), and cell composition (CC) [29].

### 2.6. KEGG Pathway Enrichment Analysis and Construction of a “Target-Pathway” Network

We also use Bioconductor software and RX 64 4.0.0 to perform GO functional enrichment analysis on the common target of YCWL and hyperlipemia and screened out the core pathways. After obtaining the KEGG pathway, Cytoscape 3.7.2 was used to construct a “target-pathway” network.

### 2.7. Molecular Docking

The three active ingredients with the highest degrees in the “TCM-Active Ingredients-Target” network diagram were molecularly docked with the four core proteins with the highest degrees in the PPI network. The structure of the compounds were obtained from the Zinc database (http://zinc.docking.org) and the PubChem database (https://pubchem.ncbi.nlm.nih.gov), and protein structure was obtained from the PDB database (http://www.rcsb. org) [30]. AutoDockTools 1.5.6 software was applied to process proteins as follows: separate proteins, add nonpolar hydrogen, calculate the Gasteiger charge, and assign the AD4 type, and set all the flexible bonds of small molecule ligands to be rotatable. The receptor protein was set to rigid docking, the genetic algorithm was selected, and the maximum number of evals was set as the medium. The docking results were obtained by running autogrid4 and autodock4, by which the binding energies were revealed. And Discovery Studio 4.5 software was used to visualize the results from the molecular docking analyses to obtain 2D and 3D images.

### 2.8. Molecular Dynamics Simulation

The amber18 software package was used to perform molecular dynamics simulation on the protein-active ingredients obtained by molecular docking. The protein uses the ff14SB force field parameter, and the active ingredients used the gaff general force field parameter and the ANTECHAMBER module to calculate its AM1-BCC atomic charge. Loading the protein, active ingredients were added into the leap module, and hydrogen atoms and antagonist ions to neutralize the charge were automatically added. The TIP3P explicit water model was selected, and periodic boundary conditions were set. The molecular dynamics simulation workflow includes four steps: energy minimization, heating, equilibrium, and production dynamics simulation. First, the heavy atoms of proteins (and small molecules) were confined, and 10,000 steps (including the 5000-step steepest descent method and the 5000-step conjugate gradient method) were performed on the water molecules to minimize the energy; then, within 50 ps, the system is slowly heated to 300 K. After the heating is completed, the system at 50 ps under the npt ensemble was balanced. Finally, the system is subjected to a molecular dynamics simulation of 50 ns (a total of 25,000 steps) under the npt ensemble, with a time step of 2 fs, and the trajectory data is saved every 10 ps, and then, the CPPTRAJ module is used for the relevant analysis. The calculation of the binding free energy of active ingredients and protein is carried out using the MMPBA.py module.

## 3. Results

### 3.1. Composition and Target Screening of YCWL

After screening, a total of 63 chemical constituents of Yinchen Wuling powder were found: 7 chemical constituents of Atractylodes lancea, 15 chemical constituents of Poria, 7 of Cinnamomi ramulus, 13 of Artemisia capillaris herba, 10 of Alismatis rhizoma, and 11 of Polyporus umbellatus, and these are shown in Supplementary Information Table S1. The protein targets found in the TCMSP database were entered into the Uniprot database for normalization, and a total of 435 gene targets were obtained, including 20 gene targets in Atractylodes lancea, 23 gene targets in Poria, 57 gene targets in Cinnamomi ramulus, 317 in Artemisia capillaris herba, 8 gene targets in Alismatis rhizoma, and 10 gene targets in Polyporus umbellatus. After merging, 260 duplicate gene targets were deleted, leaving 175 unique values.

### 3.2. Hyperlipidemia Intersectional Targets and “Chinese Medicine-Disease” Database

**Figure 2:**
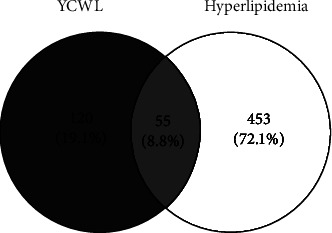
Venn diagram of YCLW (blue) and hyperlipidemia genes (yellow).

Our results reveal that the CNKI database and DisGeNet had a total of 591 targets. After removing duplicate targets, 508 targets were finally obtained. To clarify the relationship between YCWL and hyperlipidemia, EXCEL was used to merge the targets found in YCWL with hyperlipidemia, and finally, 55 intersection targets were obtained, as shown in Figure 2, and then, a Venn diagram was constructed to visualize the data (https://bioinfogp.cnb.csic.es/tools/venny).

### 3.3. Construction of a “Traditional Chinese Medicine-Active Ingredients-Target genes” Network

**Figure 3:**
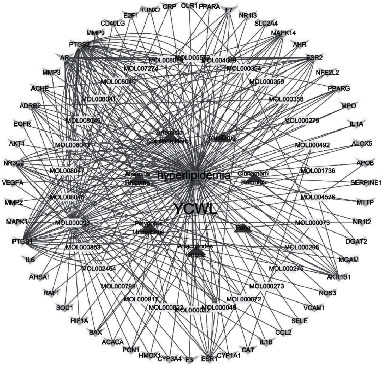
“TCM-Active Ingredients-Target” Network. (orange rectangle represents hyperlipidemia; blue rectangle represents YCWL; red triangle represents traditional Chinese medicine; yellow rectangle represents active ingredients; cyan-blue inverted triangle represents target genes).

Using EXCEL to incorporate the intersection of Chinese medicine targets and disease targets, we obtained 55 target genes, and these were imported into Cytoscape 3.7.2 to construct a traditional Chinese medicine-active ingredient-target network. From this, 95 nodes were obtained, and 618 interactional relationships were found. The “TCM-Active Ingredients-Target genes” network is shown in Figure 3.

By analyzing the network of the “TCM-Active Ingredients-target genes”, the five compounds with the highest degree values in YCWL were found to be: quercetin, isorhamnetin, taxifolin, demethoxycapillarisin, and artepillin A, and these are shown in Supplementary Information Table S2.

### 3.4. Construction of a PPI Network and Cluster Analysis

**Figure 4:**
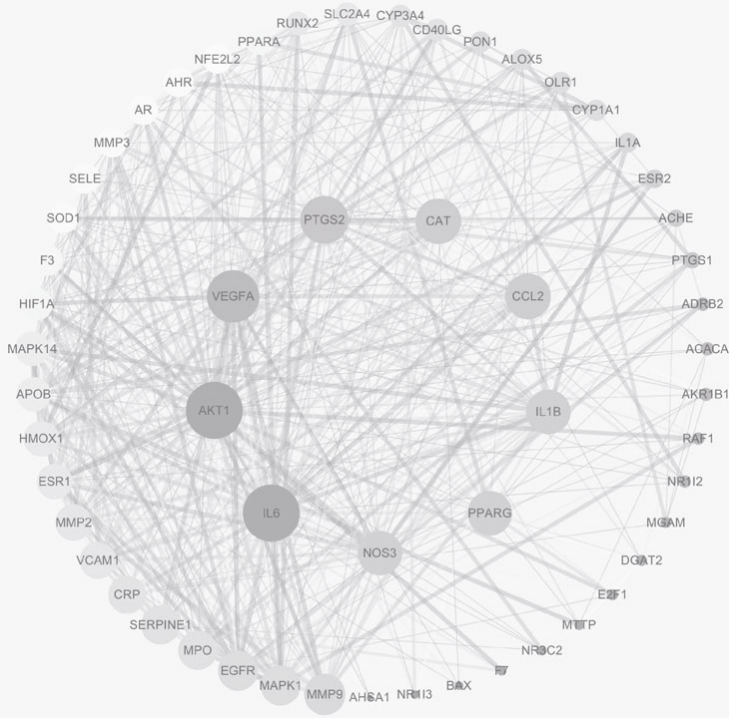
The PPI network for 55 overlapping genes (the sizes and colors of the nodes and lines are illustrated from large to small and blue to red in descending order of degree values).

**Figure 5:**
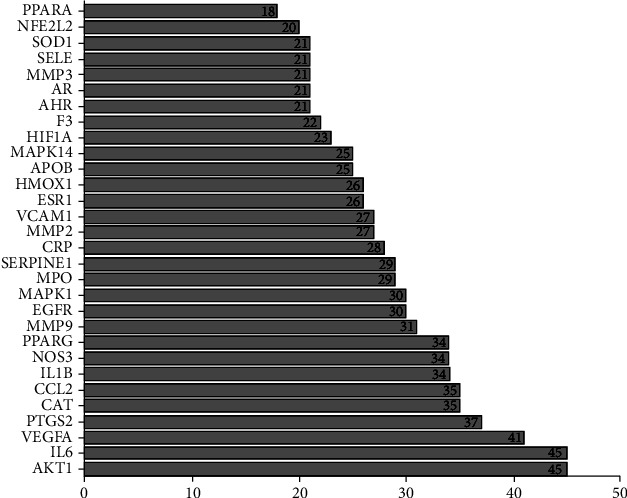
The degree value of the top 30 genes in the PPI network.

The specific names of the intersection targets were screened by EXCEL and imported into the STRING database for online PPI analysis. The interaction threshold was set to “0.4” to hide the episomal gene targets. The “edge” in the figure represents the extent of association between the intersectional genes and shows the degree of association. Thus, the greater the association, the greater the chance that the node represents the intersectional gene. There were a total of 55 nodes and 553 edges, as shown in Figure 4. PPI results showed that the top 4 were AKT1 (RAC-alpha serine/threonine-protein kinase, degree = 45), IL6 (Interleukin 6, degree = 45), VEGFA (vascular endothelial growth factor A, degree = 41), and PTGS2 (prostaglandin-endoperoxide synthase 2, degree = 37). The PPI network is shown in Figure 4, and the degree value of the top 30 genes in the PPI network is shown in Figure 5.

**Figure 6:**
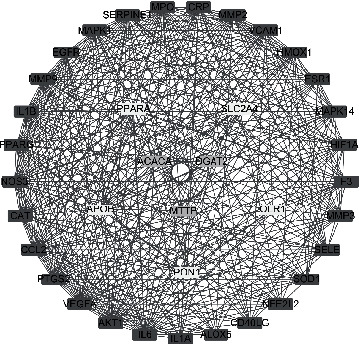
Clusters of the YCLW-hyperlipidemia network (red represents clusters 1, yellow represents clusters 2, and green represents clusters 3).

The YCLW target network clustering analysis used the same method as the PPI network clustering analysis to obtain three different clusters. The clusters of the YCLW-Hyperlipidemia is shown in Figure 6.

### 3.5. GO Enrichment Analysis

The results showed that the targets were mainly enriched in response to oxygen levels, nutrient levels, reactive oxygen species, metabolic processes, and responses to hypoxia, with a negative regulation of the apoptotic signaling pathway. Their main molecular functions were associated with the organization of vesicle lumens, membrane rafts, membrane microdomains, caveola, and membrane region, and they were mainly enriched in relation to nuclear receptor activity, transcription factor activity, direct ligand regulated sequence-specific DNA binding, steroid hormone receptor activity, heme binding, tetrapyrrole binding, and other biological processes. The top five enrichment results from each GO analysis are shown in Supplementary Information Table S3.

### 3.6. KEGG Pathway Enrichment Analysis and “Pathway-Target” Network Diagram

**Figure 7:**
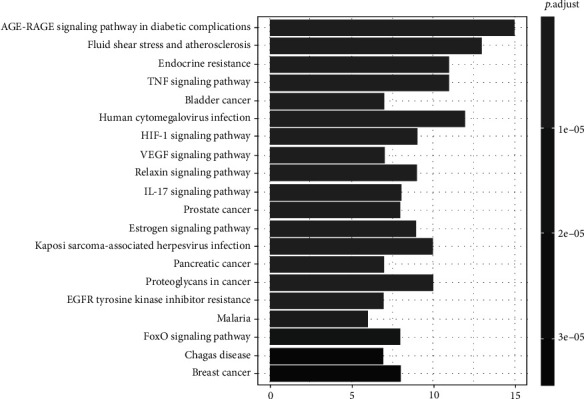
KEGG enrichment analysis. The length represents the number of target genes, and the color represents the level of significance.

**Figure 8:**
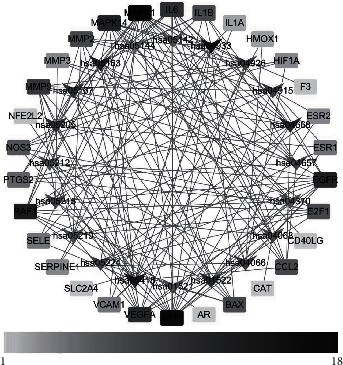
Pathway-target” network (the triangle represents the pathway，Rectangle represents the target.)

The KEGG results showed that the targets were mainly enriched in the following: the AGE-RAGE (advanced glycation end products-receptor for advanced glycation end products) signaling pathway involving diabetic complications, fluid shear stress and atherosclerosis, endocrine resistance, the TNF signaling pathway, bladder cancer, human cytomegalovirus infection, the HIF-1 signaling pathway, and the VEGF signaling pathway, and these represented 123 articles. The results of enrichment analysis from the KEGG pathway are shown in Figure 7, and the “Pathway-target” network is shown in Figure 8.

### 3.7. Molecular Docking Results

We used Autodock software to dock at the molecular level the three active ingredients (isorhamnetin, quercetin, and taxifolin) having the highest degree values with the four core proteins within the PPI network (AKT1, IL6, PTGS2, and VEGFA). The more stable the binding between the ligand and the receptor, the lower the binding energy of the two, and our results showed that the binding energy of the active ingredients and the four core proteins were all ≤-7 kcal/mol, the lowest binding free energy was PTGS2-quercetin (−11.08 kcal/mol), and the main forces involved were van der Waals forces, hydrophobic forces, and carbon-hydrogen bonds.

This was followed by PTGS2-taxifolin (-10.5 kcal/mol), and there were pi-alkyl hydrophobic forces between LYS1137 and taxifolin; the conventional hydrogen bond with THR 331, LYS1137, CYS 1047, and the carbon hydrogen bond with THR549. Then, PTGS2-isorhamnetin (-10.14 kcal/mol) and the carbon hydrogen bond with THR549 and GLY 1135 were imported into Discovery Studio 4.5 software for visual optimization. Docking scores of isorhamnetin, quercetin, and taxifolin with potential targets is shown in Supplementary Information Table S4.

**Figure 9:**
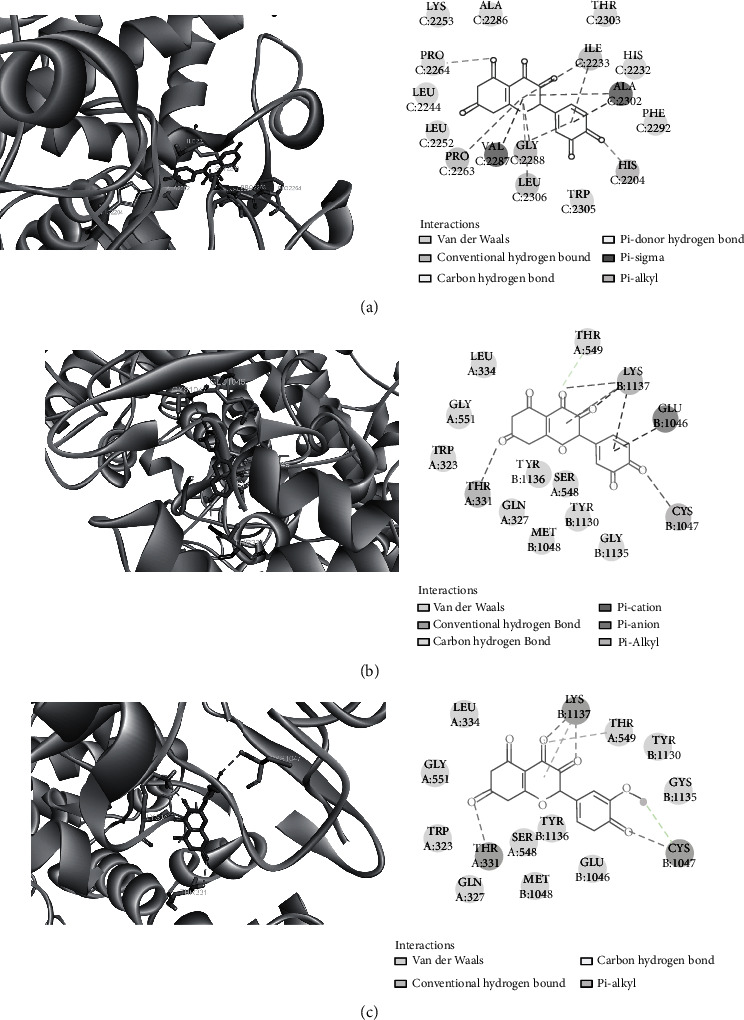
Molecular docking diagram. Molecular models of the binding of quercetin, taxifolin, and isorhamnetin with PTGS2, the results shown as 3D and 2D diagrams. (a) PTGS2-quercetin (-11.08 kcal/mol), (b) PTGS2-taxifolin (-10.5 kcal/mol), and (c) PTGS2-isorhamnetin (-10.14 kcal/mol).

The molecular docking diagram is shown in Figure 9.

### 3.8. RMSD, RMSF, and ROG Results in Molecular Dynamics Simulation

**Figure 10:**
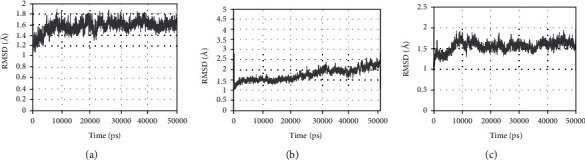
RMSD plot during molecular dynamics simulations. (a) The RMSD of PTGS2-quercetin. (b) The RMSD of PTGS2-taxifolin. (c) The RMSD of PTGS2-isorhamnetin.

The rmsd curve represents the fluctuations in protein conformation. It can be seen from Figure 10 that rmsd has certain fluctuations in the early stage. However, PTGS2-quercetin and PTGS2-isorhamnetin stabilized after 10 nm, while PTGS2-taxifolin stabilized after 30 nm. This indicates that after the small molecule ligand is combined with the protein, the conformation of the protein will not change significantly, and the combination of the two is relatively stable.

**Figure 11:**
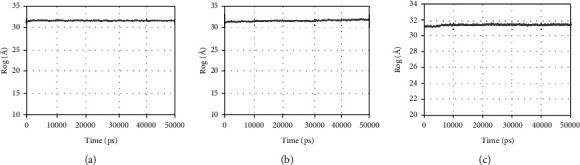
Rog plot during molecular dynamics simulations. (a) The Rog of PTGS2-quercetin. (b) The Rog of PTGS2-taxifolin. (c) The Rog of PTGS2-isorhamnetin.

The gyration radius curve represents the compactness of the overall structure of the protein. It can be seen from Figure 11 that PTGS2-quercetin, PTGS2-isorhamnetin, and PTGS2-taxifolin have stable gyration radii. This result is consistent with the rmsd result, meaning that the protein conformation is stable and is compactly folded. Furthermore, the binding of small molecules does not affect protein stability.

**Figure 12:**
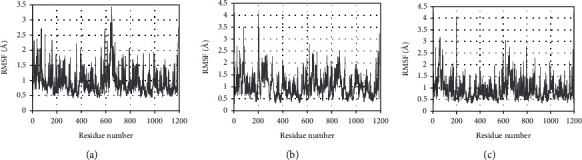
RMSF plot during molecular dynamics simulations. (a) The RMSF of PTGS2-quercetin. (b) The RMSF of PTGS2-taxifolin. (c) The RMSF of PTGS2-isorhamnetin.

The rmsf curve represents the fluctuation of the protein amino acid residues. It can be seen from Figure 12 that the results of PTGS2-quercetin, PTGS2-isorhamnetin, and PTGS2-taxifolin all show that the middle region of the protein has greater residue flexibility than other regions and this region is a loop-based polypeptide chain, and so is more flexible.

### 3.9. Free Energy of Binding and Interacting Residue Results in Molecular Dynamics Simulation

We take the trajectory of the rmsd stationary phase (10-50 ns) of PTGS2-quercetin and calculate the binding free energy. From this result, it can be seen that the binding free energy between the two is -29.5 kcal/mol. Van der Waals potential (-44 kcal/mol) and electrostatic interaction (-24 kcal/mol) are conducive to the combination of the two, while the polar solvation (EGB, 44 kcal/mol) is not conducive to the interaction of the two.

We take the trajectory of the rmsd stationary phase (30-50 ns) of PTGS2-taxifolin and calculate the binding free energy. The result shows that the binding free energy of the two is -32.9 kcal/mol. Van der Waals potential (-41 kcal/mol) and electrostatic interaction (-44 kcal/mol) are conducive to the combination of the two, while the polar solvation (EGB, 58 kcal/mol) is not conducive to the interaction of the two.

We take the trajectory of the rmsd plateau (25-50 ns) of PTGS2-isorhamnetin and calculate the binding free energy. The results show that the combination of the two are general, and the binding free energy is -32 kcal/mol. Van der Waals potential (-43.2 kcal/mol) and electrostatic interaction (-12.3 kcal/mol) are conducive to the combination of the two, while the polar solvation (EGB, 28.6 kcal/mol) is not conducive to the interaction of the two. Free energies of the binding are shown in the Supplementary Information Table S5-7.

**Figure 13:**
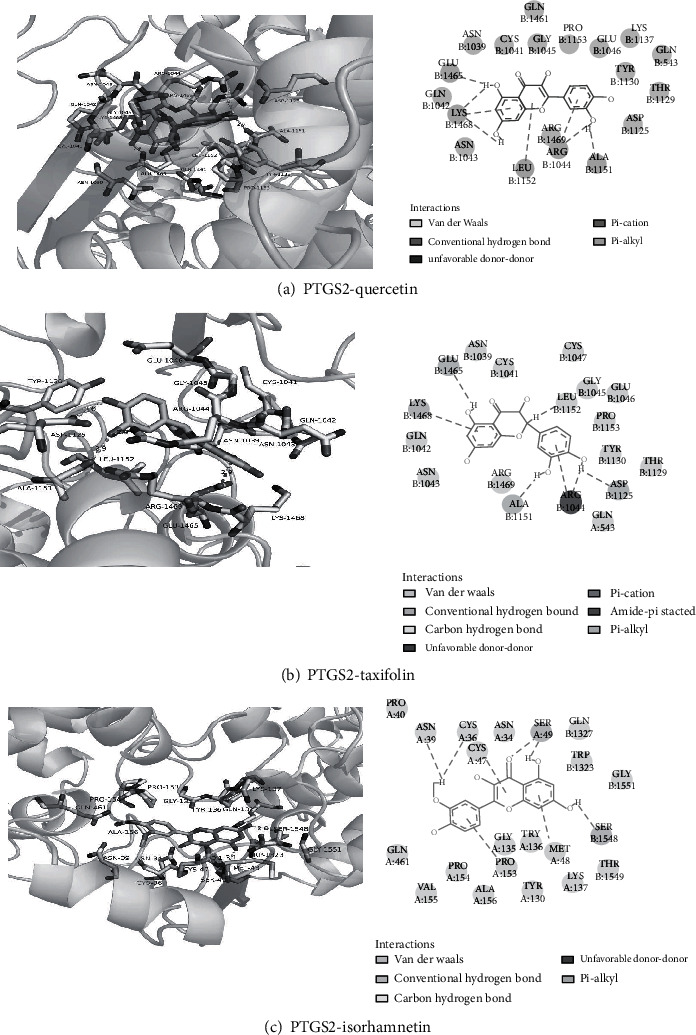
(a) 3D and 2D diagrams of PTGS2-quercetin interaction. (b) 3D and 2D diagrams of PTGS2- taxifolin interaction. (c) 3D and 2D diagrams of PTGS2-isorhamnetin interactions.

It can be seen from Figure 13(a) that the PTGS2-quercetin interaction binding site forms a relatively hydrophilic environment with strong hydrophilicity. The residues that interact with quercetin are mainly hydrophilic residues, such as Asp1125, Glu1046, and Lys1468. Quercetin can form hydrogen bonds with Glu1465, Arg1044, and Lys1468.

It can be seen from Figure 13(b) that the PTGS2-taxifolin interaction binding site forms a relatively hydrophilic environment with strong hydrophilicity. The residues that interact with taxifolin are mainly hydrophilic residues, such as Arg1469, Glu1465, and Lys1468. Taxifolin can form hydrogen bonds with Glu1465, Ala1151, Arg1044, and Asp1125.

It can be seen from Figure 13(c) that the PTGS2-isorhamnetin binding site forms a relatively hydrophobic environment with strong hydrophobicity. The residues that interact with isorhamnetin are mainly hydrophobic residues, such as Tyr136, Val155, and Trp1323. Isorhamnetin can form hydrogen bonds with Ser49, Met48, and SER1548.

## 4. Discussion

In this study, based on network pharmacology, we found that YCWL principally regulates hyperlipidemia through quercetin, isorhamnetin, and taxifolin as well as other components. Studies have found that quercetin can reduce blood lipid levels by upregulating the expression of ABCA1, ABCG1, and other proteins and by increasing the cholesterol binding affinity of HDL and APOA1 [31]. Low concentrations of quercetin can also reduce the secretion of PCSK9, thereby stimulating the uptake of low-density lipoprotein and reducing its bioavailability [32]. Previous experiments have found that quercetin can also reduce the levels of TC, TG, LDL, and FFA in hyperlipidemic rats and can regulate HDL back to near normal levels, as well as lowering blood sugar and improving hypertension [33, 34]. Isorhamnetin has been found to have anti-inflammatory and antioxidative effects and its mechanism of action is thought to involve the inhibition of NF-*κ*B and block the Toll-like receptor 4 signaling pathway. It can also reduce the production of reactive oxygen species and alleviate the inflammatory response of mouse microglia induced by lipopolysaccharide. Furthermore, Zhang et al. [35] observed a protective effect of ISO on the oxidative stress injury model in vascular endothelial cells, and the results showed that isorhamnetin has the ability to prevent apoptosis and promote the proliferation of damaged vascular endothelial cells. Furthermore, Dong et al. found that isorhamnetin also possesses antioxidant and antiapoptotic effects in cardiomyocytes and this works by activation of the Nrf2 pathway [36]. Taxifolin produces its pharmacological effects by reducing inflammation and tumor oxidative stress, as well as affecting the cardiovascular system [37, 38]. It inhibits the activity of oxidases reducing excessive production of reactive oxygen species (ROS). As it can reduce oxidative stress damage, it can improve rat ischemia-reperfusion injury by inhibiting the mitochondrial apoptosis pathway [39]. Sun et al. found that TAX can improve diabetic cardiomyopathy by regulating oxidative stress and apoptosis in cardiomyocytes [40].

The results from the PPI analysis showed that the targets for YCWL relating to the treatment of hyperlipidemia mainly involved AKT1, IL6, VEGFA, and PTGS2. There are three known subtypes of AKT, and it can play a regulatory role in glucose homeostasis, protein synthesis, lipid metabolism, cell proliferation, and survival. When AKT is activated by PI3K, it can then phosphorylate downstream substrates and these mechanisms are involved in the regulation of cell apoptosis, lipid metabolism, and cell proliferation. Studies have also shown that insulin secretions after food intake results in the activation of the PI3K/AKT signaling pathway [41] thus increasing the utilization of glucose, increasing lipid deposition in the body, and reducing free fatty acids in the circulation. Activation of the PI3K/AKT pathway can also reduce obesity and insulin resistance. Importantly, when PI3K/AKT is overexpressed or mutated, it can cause obesity, cancer, and other diseases. IL6 is a proinflammatory cytokine, which can upregulate the expression of adhesion molecules, thereby enhancing the inflammatory response and can also affect the rate of lipid metabolisms, specifically of TCs and TGs. It also plays an important role in inflammation, atherosclerosis, and thrombosis [42]. There is a close relationship between dyslipidemia and IL6 and insulin resistance where insulin resistance, or insufficient insulin secretion, can promote the secretion of large amounts of IL6 by pancreatic islet cells, thereby over activating B and T lymphocytes, as well as interacting with other cytokines. IL6, combined with cytotoxicity produced by effector cells, leads to the death of pancreatic *β*-cells and aggravates insulin resistance. Therefore, it regulates blood lipids and reduces the release of inflammatory mediators, which can effectively reduce the occurrence of atherosclerosis and acute myocardial infarction [43]. VEGFA is an important factor that regulates vascular endothelial cells and plays a role in promoting their survival and enhancing vascular permeability. Studies have shown that vascular remodeling during atherosclerosis is related to the expression of VEGFA [44]. PTGS is a cyclooxygenase (COX), which includes two isozymes PTGS1 and PTGS2. PTGS2 is involved in various signal transduction pathways involved in pain, inflammation, tumor neoplasia, atherosclerosis, and coronary heart disease [45, 46]. It has recently been found that COX-2 can induce the synthesis of VEGF, promote the formation of new blood vessels, and can promote the pathogenesis and expansion of atherosclerotic plaques [47].

Our molecular results showed that the binding energies of the three active ingredients, and the four core proteins were all ≤-7 kcal/mol, indicating that the abovementioned active ingredients had good binding for their target, while the binding energy of PTGS2-quercetin (-11.08 kcal/mol) was the lowest. This indicates that it may be the core target and active ingredient of YCWL when treating hyperlipidemia. In addition, molecular dynamics simulation results show that PTGS2-quercetin, PTGS2-isorhamnetin, and PTGS2-taxifolin bind more stably, and their binding free energies are PTGS2-quercetin (-29.5 kcal/mol), PTGS2-isorhamnetin (-32 kcal/mol), and PTGS2-taxifolin (-32.9 kcal/mol).

GO enrichment analysis showed that YCWL mainly regulates hyperlipidemia through biological processes such as response to oxygen levels, nutrient levels, reactive oxygen species, and other metabolic process, including hypoxia, and can also negatively regulate the apoptotic signaling pathway. KEGG pathway enrichment analysis showed that the main pathways that regulate hyperlipidemia are the AGE-RAGE signaling pathway in diabetic complications, fluid shear stress, and atherosclerosis, the TNF signaling pathway, and the HIF-1 signaling pathway. Studies have found that a high-fat diet and high blood sugar will increase the formation and accumulation of AGE. Increased AGE may cause inflammation, oxidative stress, and the activation of fat storage [48]. The activation of the AGE-RAGE signaling pathway can induce the production of NF-*κ*B, VCAM-1, tissue factor, and inflammatory factors and is one of the most relevant signaling pathways associated with chronic diseases such as diabetes, atherosclerosis, and cancer [49]. The HIF-1 signaling pathway can inhibit the catabolism of fatty acids by inhibiting long-chain acyl-CoA dehydrogenase and medium-chain acyl-CoA dehydrogenase, leading to a reduction in reactive oxygen species and fat accumulation [50]. Mylonis et al. found that under hypoxic conditions, HIF can upregulate glycolytic pathways and reduce mitochondrial function. It can also induce fatty acid uptake, synthesis, and storage genes and enhance fat production [51]. Activation of the TNF-*α* pathway can induce oxidative stress and increase transcytosis of LDLs across endothelial cells and promote the development of atherosclerosis [52]. Fluid shear stress and the atherosclerotic pathway play important roles in the development of hyperlipidemia and its progression to atherosclerosis. Studies have also shown that this shear stress can be divided into two types, a temporal gradient within the shear and a steady shear. The temporal gradient within the shear can upregulate the expression of proinflammatory factors and apoptotic genes in vascular endothelial cells, which can induce inflammation, cell migration, and apoptosis and ultimately lead to the formation of atherosclerosis. However, steady shear stress maintains upregulated genes involved in antiatherosclerotic effects such as manganese superoxide dismutase and cyclooxygenase 2 [53, 54].

Based on network pharmacology and molecular docking technologies and using PPI, GO, and KEGG analyses of novel targets, we were able to predict a role for YCWL in the regulation of the AGE-RAGE signaling pathway during diabetic complications, and this regulation was associated with AKT1, IL6, VEGFA, PTGS2, and other targets. We further found that fluid shear stress and atherosclerosis, the TNF signaling pathway, the HIF-1 signaling pathway, and other signaling pathways play a role in the regulation of hyperlipidemia. Therefore, this work has clearly highlighted some of the multiple components found within YCWL and found some of its targets which may be involved in its beneficial effects upon hyperlipidemia. These data supports or views that additional *in vitro* and *in vivo* studies are required to further elucidate its function and mechanism of action.

## Data Availability

We have presented all our main data in the form of figures and an additional file. The data will be available upon request.

## Abbreviations

TC: Total cholesterol
TG: Triglycerides
LDL-C: Low-density lipoprotein cholesterol
HDL-C: High-density lipoprotein cholesterol
YCWL: Yinchen Wuling powder
PPAR: Peroxisome proliferator-activated receptors
GLUT4: Glucose transporter 4
IRS-1: Insulin receptor substrate 1
AMPL: AMP-activated kinase
FINS: Fasting insulin
FBG: Fasting blood glucose
OB: Oral bioavailability
DL: Drug-likeness
PPI: Protein-protein interaction network
BP: Biological process
MF: Molecular function
CC: Cell composition
AKT1: RAC-alpha serine/threonine-protein kinase
IL6: Interleukin 6
VEGFA: Vascular endothelial growth factor A
PTGS2: Prostaglandin-endoperoxide synthase 2
ROS: Reactive oxygen species
COX: Cyclooxygenase.

## Supplementary Materials

Supplementary Materials Supplementary Information Table S1: active ingredients found in YCWL. Supplementary information Table S2: top five active ingredients found in YCWL. Supplementary information Table S3: top five enrichment results from each GO analysis. Supplementary information Table S4: molecular docking scores. Supplementary information Table S5: free energies of binding for PTGS2-quercetin. Supplementary information Table S6: free energies of binding for PTGS2-taxifolin. Supplementary information Table S7: free energies of binding for PTGS2-isorhamnetin.
