# Supplementary material for: Exploring the Molecular Mechanism of Action of Yinchen Wuling Powder for the Treatment of Hyperlipidemia, Using Network Pharmacology, Molecular Docking, and Molecular Dynamics Simulation
Source: Biomed Res Int. 2021 Oct 28;2021:9965906. doi: 10.1155/2021/9965906 (PMC8568510; doi:10.1155/2021/9965906)
Supplement: Supplementary Materials — Supplementary Information Table S1: active ingredients found in YCWL. Supplementary information Table S2: top five active ingredients found in YCWL. Supplementary information Table S3: top five enrichment results from each GO analysis. Supplementary information Table S4: molecular docking scores. Supplementary information Table S5: free energies of binding for PTGS2-quercetin. Supplementary information Table S6: free energies of binding for PTGS2-taxifolin. Supplementary information Table S7: free energies of binding for PTGS2-isorhamnetin. [file 9965906.f1.zip › 9965906.f1.docx]

**Supplementary information table S1: Active ingredients found in YCWL**

| Drug | Mol ID | Molecule Name | OB (%) | DL |
| --- | --- | --- | --- | --- |
| Atractylodes lancea | MOL000020 | 12-senecioyl-2E,8E,10E-atractylentriol | 62.4 | 0.22 |
| Atractylodes lancea | MOL000021 | 14-acetyl-12-senecioyl-2E,8E,10E-atractylentriol | 60.31 | 0.31 |
| Atractylodes lancea | MOL000022 | 14-acetyl-12-senecioyl-2E,8Z,10E-atractylentriol | 63.37 | 0.3 |
| Atractylodes lancea | MOL000028 | α-Amyrin | 39.51 | 0.76 |
| Atractylodes lancea | MOL000033 | (3S,8S,9S,10R,13R,14S,17R)-10,13-dimethyl-17-[(2R,5S)-5-propan-2-yloctan-2-yl]-2,3,4,7,8,9,11,12,14,15,16,17-dodecahydro-1H-cyclopenta[a]phenanthren-3-ol | 36.23 | 0.78 |
| Atractylodes lancea | MOL000049 | 3β-acetoxyatractylone | 54.07 | 0.22 |
| Atractylodes lancea | MOL000072 | 8β-ethoxy atractylenolide Ⅲ | 35.95 | 0.21 |
| Poria | MOL000273 | (2R)-2-[(3S,5R,10S,13R,14R,16R,17R)-3,16-dihydroxy-4,4,10,13,14-pentamethyl-2,3,5,6,12,15,16,17-octahydro-1H-cyclopenta[a]phenanthren-17-yl]-6-methylhept-5-enoic acid | 30.93 | 0.81 |
| Poria | MOL000275 | trametenolic acid | 38.71 | 0.8 |
| Poria | MOL000276 | 7,9(11)-dehydropachymic acid | 35.11 | 0.81 |
| Poria | MOL000279 | Cerevisterol | 37.96 | 0.77 |
| Poria | MOL000280 | (2R)-2-[(3S,5R,10S,13R,14R,16R,17R)-3,16-dihydroxy-4,4,10,13,14-pentamethyl-2,3,5,6,12,15,16,17-octahydro-1H-cyclopenta[a]phenanthren-17-yl]-5-isopropyl-hex-5-enoic acid | 31.07 | 0.82 |
| Poria | MOL000282 | ergosta-7,22E-dien-3beta-ol | 43.51 | 0.72 |
| Poria | MOL000283 | Ergosterol peroxide | 40.36 | 0.81 |
| Poria | MOL000285 | (2R)-2-[(5R,10S,13R,14R,16R,17R)-16-hydroxy-3-keto-4,4,10,13,14-pentamethyl-1,2,5,6,12,15,16,17-octahydrocyclopenta[a]phenanthren-17-yl]-5-isopropyl-hex-5-enoic acid | 38.26 | 0.82 |
| Poria | MOL000287 | 3beta-Hydroxy-24-methylene-8-lanostene-21-oic acid | 38.7 | 0.81 |
| Poria | MOL000289 | pachymic acid | 33.63 | 0.81 |
| Poria | MOL000290 | Poricoic acid A | 30.61 | 0.76 |
| Poria | MOL000291 | Poricoic acid B | 30.52 | 0.75 |
| Poria | MOL000292 | poricoic acid C | 38.15 | 0.75 |
| Poria | MOL000296 | hederagenin | 36.91 | 0.75 |
| Poria | MOL000300 | dehydroeburicoic acid | 44.17 | 0.83 |
| Cinnamomi Ramulus | MOL001736 | (-)-taxifolin | 60.51 | 0.27 |
| Cinnamomi Ramulus | MOL000358 | beta-sitosterol | 36.91 | 0.75 |
| Cinnamomi Ramulus | MOL000359 | sitosterol | 36.91 | 0.75 |
| Cinnamomi Ramulus | MOL000492 | (+)-catechin | 54.83 | 0.24 |
| Cinnamomi Ramulus | MOL000073 | ent-Epicatechin | 48.96 | 0.24 |
| Cinnamomi Ramulus | MOL004576 | taxifolin | 57.84 | 0.27 |
| Cinnamomi Ramulus | MOL011169 | Peroxyergosterol | 44.39 | 0.82 |
| Artemisia Capillaris Herba | MOL000354 | isorhamnetin | 49.6 | 0.31 |
| Artemisia Capillaris Herba | MOL000358 | beta-sitosterol | 36.91 | 0.75 |
| Artemisia Capillaris Herba | MOL004609 | Areapillin | 48.96 | 0.41 |
| Artemisia Capillaris Herba | MOL005573 | Genkwanin | 37.13 | 0.24 |
| Artemisia Capillaris Herba | MOL007274 | Skrofulein | 30.35 | 0.3 |
| Artemisia Capillaris Herba | MOL008039 | Isoarcapillin | 57.4 | 0.41 |
| Artemisia Capillaris Herba | MOL008040 | Eupalitin | 46.11 | 0.33 |
| Artemisia Capillaris Herba | MOL008041 | Eupatolitin | 42.55 | 0.37 |
| Artemisia Capillaris Herba | MOL008043 | capillarisin | 57.56 | 0.31 |
| Artemisia Capillaris Herba | MOL008045 | 4'-Methylcapillarisin | 72.18 | 0.35 |
| Artemisia Capillaris Herba | MOL008046 | Demethoxycapillarisin | 52.33 | 0.25 |
| Artemisia Capillaris Herba | MOL008047 | Artepillin A | 68.32 | 0.24 |
| Artemisia Capillaris Herba | MOL000098 | quercetin | 46.43 | 0.28 |
| Alismatis Rhizoma | MOL000359 | sitosterol | 36.91 | 0.75 |
| Alismatis Rhizoma | MOL000830 | Alisol B | 34.47 | 0.82 |
| Alismatis Rhizoma | MOL000831 | Alisol B monoacetate | 35.58 | 0.81 |
| Alismatis Rhizoma | MOL000832 | alisol, b,23-acetate | 32.52 | 0.82 |
| Alismatis Rhizoma | MOL000849 | 16-methoxyalisol B monoacetate | 32.43 | 0.77 |
| Alismatis Rhizoma | MOL000853 | alisol B | 36.76 | 0.82 |
| Alismatis Rhizoma | MOL000854 | alisol C | 32.7 | 0.82 |
| Alismatis Rhizoma | MOL000856 | alisol C monoacetate | 33.06 | 0.83 |
| Alismatis Rhizoma | MOL002464 | 1-Monolinolein | 37.18 | 0.3 |
| Alismatis Rhizoma | MOL000862 | [(1S,3R)-1-[(2R)-3,3-dimethyloxiran-2-yl]-3-[(5R,8S,9S,10S,11S,14R)-11-hydroxy-4,4,8,10,14-pentamethyl-3-oxo-1,2,5,6,7,9,11,12,15,16-decahydrocyclopenta[a]phenanthren-17-yl ]butyl] acetate | 35.58 | 0.81 |
| Polyporus Umbellatus | MOL000279 | Cerevisterol | 37.96 | 0.77 |
| Polyporus Umbellatus | MOL000282 | ergosta-7,22E-dien-3beta-ol | 43.51 | 0.72 |
| Polyporus Umbellatus | MOL000796 | (22e,24r)-ergosta-6-en-3beta,5alpha,6beta-triol | 30.2 | 0.76 |
| Polyporus Umbellatus | MOL000797 | (22e,24r)-ergosta-7,22-dien-3-one | 44.88 | 0.72 |
| Polyporus Umbellatus | MOL000798 | ergosta-7,22-diene-3-ol | 43.51 | 0.72 |
| Polyporus Umbellatus | MOL000801 | 5alpha,8alpha-epidioxy-(22e,24r)-ergosta-6,22-dien-3beta-ol | 44.39 | 0.82 |
| Polyporus Umbellatus | MOL011169 | Peroxyergosterol | 44.39 | 0.82 |
| Polyporus Umbellatus | MOL000816 | ergosta-7,22-dien-3-one | 44.88 | 0.72 |
| Polyporus Umbellatus | MOL000817 | ergosta-5,7,22-trien-3-ol | 46.18 | 0.72 |
| Polyporus Umbellatus | MOL000820 | polyporusterone E | 45.71 | 0.85 |
| Polyporus Umbellatus | MOL000822 | polyporusterone G | 33.43 | 0.81 |
