# Supplementary material for: Exploring the Molecular Mechanism of Action of Yinchen Wuling Powder for the Treatment of Hyperlipidemia, Using Network Pharmacology, Molecular Docking, and Molecular Dynamics Simulation
Source: Biomed Res Int. 2021 Oct 28;2021:9965906. doi: 10.1155/2021/9965906 (PMC8568510; doi:10.1155/2021/9965906)
Supplement: Supplementary Materials — Supplementary Information Table S1: active ingredients found in YCWL. Supplementary information Table S2: top five active ingredients found in YCWL. Supplementary information Table S3: top five enrichment results from each GO analysis. Supplementary information Table S4: molecular docking scores. Supplementary information Table S5: free energies of binding for PTGS2-quercetin. Supplementary information Table S6: free energies of binding for PTGS2-taxifolin. Supplementary information Table S7: free energies of binding for PTGS2-isorhamnetin. [file 9965906.f1.zip › 9965906.f2.docx]

**Supplementary information table S2: Top five active ingredients found in YCWL**

| **Type** | **ID** | **name** | **Degree** |
| --- | --- | --- | --- |
| Artemisia Capillaris Thunb | MOL000098 | quercetin | 48 |
| Artemisia Capillaris Thunb | MOL000354 | isorhamnetin | 12 |
| Cinnamomi Ramulus | MOL004576 | taxifolin | 7 |
| Artemisia Capillaris Thunb | MOL008046 | Demethoxycapillarisin | 7 |
| Artemisia Capillaris Thunb | MOL008047 | Artepillin A | 7 |
