# Supplementary material for: Exploring the Molecular Mechanism of Action of Yinchen Wuling Powder for the Treatment of Hyperlipidemia, Using Network Pharmacology, Molecular Docking, and Molecular Dynamics Simulation
Source: Biomed Res Int. 2021 Oct 28;2021:9965906. doi: 10.1155/2021/9965906 (PMC8568510; doi:10.1155/2021/9965906)
Supplement: Supplementary Materials — Supplementary Information Table S1: active ingredients found in YCWL. Supplementary information Table S2: top five active ingredients found in YCWL. Supplementary information Table S3: top five enrichment results from each GO analysis. Supplementary information Table S4: molecular docking scores. Supplementary information Table S5: free energies of binding for PTGS2-quercetin. Supplementary information Table S6: free energies of binding for PTGS2-taxifolin. Supplementary information Table S7: free energies of binding for PTGS2-isorhamnetin. [file 9965906.f1.zip › 9965906.f3.docx]

Supplementary information table S3 ：Top five Enrichment Results from each Go analysis

| Type | ID | Description | p value | Count |
| --- | --- | --- | --- | --- |
| BP | GO:0070482 | response to oxygen levels | 2.21E-14 | 16 |
| BP | GO:0031667 | response to nutrient levels | 5.18E-14 | 17 |
| BP | GO:0072593 | reactive oxygen species metabolic process | 8.45E-14 | 14 |
| BP | GO:0001666 | response to hypoxia | 1.08E-13 | 15 |
| BP | GO:2001234 | negative regulation of apoptotic signaling pathway | 1.27E-13 | 13 |
| CC | GO:0031983 | vesicle lumen | 4.39E-07 | 9 |
| CC | GO:0045121 | membrane raft | 2.83E-06 | 8 |
| CC | GO:0098857 | membrane microdomain | 2.90E-06 | 8 |
| CC | GO:0005901 | caveola | 3.15E-06 | 5 |
| CC | GO:0098589 | membrane region | 3.82E-06 | 8 |
| MF | GO:0004879 | nuclear receptor activity | 1.71E-12 | 8 |
| MF | GO:0098531 | transcription factor activity, direct ligand regulated sequence-specific DNA binding | 1.71E-12 | 8 |
| MF | GO:0003707 | steroid hormone receptor activity | 7.54E-12 | 8 |
| MF | GO:0020037 | heme binding | 9.71E-09 | 8 |
| MF | GO:0046906 | tetrapyrrole binding | 1.70E-08 | 8 |
