# Supplementary material for: Exploring the Molecular Mechanism of Action of Yinchen Wuling Powder for the Treatment of Hyperlipidemia, Using Network Pharmacology, Molecular Docking, and Molecular Dynamics Simulation
Source: Biomed Res Int. 2021 Oct 28;2021:9965906. doi: 10.1155/2021/9965906 (PMC8568510; doi:10.1155/2021/9965906)
Supplement: Supplementary Materials — Supplementary Information Table S1: active ingredients found in YCWL. Supplementary information Table S2: top five active ingredients found in YCWL. Supplementary information Table S3: top five enrichment results from each GO analysis. Supplementary information Table S4: molecular docking scores. Supplementary information Table S5: free energies of binding for PTGS2-quercetin. Supplementary information Table S6: free energies of binding for PTGS2-taxifolin. Supplementary information Table S7: free energies of binding for PTGS2-isorhamnetin. [file 9965906.f1.zip › 9965906.f4.docx]

Supplementary information table S4: Molecular Docking scores

| Number | proteins | PDB ID | Compound | Binding energies(kcal/mol) | Grid box size |
| --- | --- | --- | --- | --- | --- |
| 1 | AKT1 | 5AAR | isorhamnetin | -7.57 | 126*126*126 |
|  |  |  | quercetin | -7.67 | 126*126*126 |
|  |  |  | taxifolin | -7.77 | 126*126*126 |
| 2 | IL6 | 4O9H | isorhamnetin | -8.21 | 126*126*126 |
|  |  |  | quercetin | -9.41 | 126*126*126 |
|  |  |  | taxifolin | -9.48 | 126*126*126 |
| 3 | PTGS2 | 1PXX | isorhamnetin | -10.14 | 126*126*126 |
|  |  |  | quercetin | -11.08 | 126*126*126 |
|  |  |  | taxifolin | -10.5 | 126*126*126 |
| 4 | VEGFA | 5DN2 | isorhamnetin | -8.83 | 126*126*126 |
|  |  |  | quercetin | -10.06 | 126*126*126 |
|  |  |  | taxifolin | -10.13 | 126*126*126 |
