# Supplementary material for: Exploring the Molecular Mechanism of Action of Yinchen Wuling Powder for the Treatment of Hyperlipidemia, Using Network Pharmacology, Molecular Docking, and Molecular Dynamics Simulation
Source: Biomed Res Int. 2021 Oct 28;2021:9965906. doi: 10.1155/2021/9965906 (PMC8568510; doi:10.1155/2021/9965906)
Supplement: Supplementary Materials — Supplementary Information Table S1: active ingredients found in YCWL. Supplementary information Table S2: top five active ingredients found in YCWL. Supplementary information Table S3: top five enrichment results from each GO analysis. Supplementary information Table S4: molecular docking scores. Supplementary information Table S5: free energies of binding for PTGS2-quercetin. Supplementary information Table S6: free energies of binding for PTGS2-taxifolin. Supplementary information Table S7: free energies of binding for PTGS2-isorhamnetin. [file 9965906.f1.zip › 9965906.f5.docx]

| Energy Component | Average | Std. Dev. | Std. Err. of Mean |
| --- | --- | --- | --- |
|  |  |  |  |
| VDWAALS | -44.555 | 3.0896 | 0.6059 |
| EEL | -24.5661 | 6.0043 | 1.1775 |
| EGB | 44.5392 | 4.2624 | 0.8359 |
| ESURF | -4.9363 | 0.1167 | 0.0229 |
|  |  |  |  |
| DELTA G gas | -69.1211 | 5.4503 | 1.0689 |
| DELTA G solv | 39.6029 | 4.3002 | 0.8433 |
|  |  |  |  |
| DELTA TOTAL | -29.5182 | 3.0474 | 0.5977 |
