# Supplementary material for: Exploring the Molecular Mechanism of Action of Yinchen Wuling Powder for the Treatment of Hyperlipidemia, Using Network Pharmacology, Molecular Docking, and Molecular Dynamics Simulation
Source: Biomed Res Int. 2021 Oct 28;2021:9965906. doi: 10.1155/2021/9965906 (PMC8568510; doi:10.1155/2021/9965906)
Supplement: Supplementary Materials — Supplementary Information Table S1: active ingredients found in YCWL. Supplementary information Table S2: top five active ingredients found in YCWL. Supplementary information Table S3: top five enrichment results from each GO analysis. Supplementary information Table S4: molecular docking scores. Supplementary information Table S5: free energies of binding for PTGS2-quercetin. Supplementary information Table S6: free energies of binding for PTGS2-taxifolin. Supplementary information Table S7: free energies of binding for PTGS2-isorhamnetin. [file 9965906.f1.zip › 9965906.f6.docx]

| Energy Component | Average | Std. Dev. | Std. Err. of Mean |
| --- | --- | --- | --- |
|  |  |  |  |
| VDWAALS | -41.4817 | 3.311 | 0.6493 |
| EEL | -44.4412 | 8.6733 | 1.701 |
| EGB | 58.3977 | 5.7941 | 1.1363 |
| ESURF | -5.3817 | 0.1143 | 0.0224 |
|  |  |  |  |
| DELTA G gas | -85.9229 | 7.3437 | 1.4402 |
| DELTA G solv | 53.0161 | 5.764 | 1.1304 |
|  |  |  |  |
| DELTA TOTAL | -32.9069 | 2.5716 | 0.5043 |
