# Supplementary material for: Exploring the Molecular Mechanism of Action of Yinchen Wuling Powder for the Treatment of Hyperlipidemia, Using Network Pharmacology, Molecular Docking, and Molecular Dynamics Simulation
Source: Biomed Res Int. 2021 Oct 28;2021:9965906. doi: 10.1155/2021/9965906 (PMC8568510; doi:10.1155/2021/9965906)
Supplement: Supplementary Materials — Supplementary Information Table S1: active ingredients found in YCWL. Supplementary information Table S2: top five active ingredients found in YCWL. Supplementary information Table S3: top five enrichment results from each GO analysis. Supplementary information Table S4: molecular docking scores. Supplementary information Table S5: free energies of binding for PTGS2-quercetin. Supplementary information Table S6: free energies of binding for PTGS2-taxifolin. Supplementary information Table S7: free energies of binding for PTGS2-isorhamnetin. [file 9965906.f1.zip › 9965906.f7.docx]

| Energy Component | Average | Std. Dev. | Std. Err. of Mean |
| --- | --- | --- | --- |
|  |  |  |  |
| VDWAALS | -43.1583 | 2.2241 | 0.4362 |
| EEL | -12.2897 | 3.7593 | 0.7373 |
| EGB | 28.6491 | 2.7053 | 0.5306 |
| ESURF | -5.2108 | 0.1181 | 0.0232 |
|  |  |  |  |
| DELTA G gas | -55.448 | 3.9771 | 0.78 |
| DELTA G solv | 23.4383 | 2.6734 | 0.5243 |
|  |  |  |  |
| DELTA TOTAL | -32.0097 | 2.7986 | 0.5488 |
